# Validation and standardization of the Scale of Self-Perception of Central Auditory Processing Skills (EAPAC) - expanded

**DOI:** 10.1590/2317-1782/e20250215en

**Published:** 2026-06-19

**Authors:** Luciana Cássia de Jesus, Luciana Mendonça Alves, Luciana Macedo de Resende

**Affiliations:** 1 Programa de Pós-graduação em Ciências Fonoaudiológicas, Faculdade de Medicina, Universidade Federal de Minas Gerais – UFMG - Belo Horizonte (MG), Brasil.; 2 Departamento de Fonoaudiologia, Faculdade de Medicina, Universidade Federal de Minas Gerais – UFMG - Belo Horizonte (MG), Brasil.

**Keywords:** Hearing, Auditory Perception, Surveys and Questionnaires, Self Report, Validation Study, Cognition, Communication, Adult

## Abstract

**Purpose:**

To validate and standardize the online application of the Scale of Self-Perception of Central Auditory Processing Skills (EAPAC) – expanded for Brazilian adults.

**Methods:**

The EAPAC was expanded and answered by 1,274 Brazilian individuals aged 18 to 59 years. Of these, 116 subjects underwent neuropsychological and auditory evaluation, and 63 were selected to answer the scale at a second time point. Those with any type and degree of hearing loss, history of speech-language-hearing therapy for central auditory processing disorder, and psychiatric and/or neurological alteration were excluded. Descriptive analysis of the variables, exploratory factor analysis, and ROC curve were performed for validation and standardization. The Wilcoxon test was used to compare the responses of the subjects who answered the scale at two time points.

**Results:**

After exploratory factor analysis, five questions were excluded, as they did not correlate with the scale domains. The ROC curves, constructed considering the presence of auditory processing disorder and the result by auditory processes, did not indicate significant values. Thus, the scale presented fragile sensitivity and specificity values. There was no significant difference in the subjects’ responses to the scale between the two time points.

**Conclusion:**

The expanded scale did not present adequate psychometric standards for identifying the risk for central auditory processing disorder, but it proved useful for investigating auditory complaints in everyday tasks, thus helping to select strategies to minimize the negative impact of the difficulties mentioned.

## INTRODUCTION

Hearing is crucial for everyday professional, academic, and communicative tasks, allowing the capture and analysis of sound to generate appropriate responses^(1)^. Central auditory processing (CAP) is responsible for discriminating and interpreting these sound messages, supported by cognitive skills such as working memory, attention, and speed of information processing^(1-4)^.

CAP disorder (CAPD) occurs in the presence of normal hearing, impairing sound analysis, especially in acoustically challenging environments^(1-3)^. Although it mainly affects children, adults also show symptoms, impacting their communication and social performance, which makes it relevant to study auditory skills in this group^(1-5)^.

A study demonstrated that many adults with hearing complaints and normal audiometric thresholds are not referred for CAP evaluation. Most self-reported moderate to severe hearing problems on the Hearing Handicap Inventory for Adults – HHIA-S. More than half of the individuals who complained of hearing difficulty had CAPD^(6)^.

CAP is assessed through behavioral and electrophysiological tests. It is recommended to use self-perception questionnaires to identify the risk for CAPD, analyze the subject's perception of their health status, record the difficulties presented, and develop therapeutic strategies, since they are simple and low-cost resources^(3,5,7-9)^.

Auditory self-perception checklists correlate with auditory skills assessed through behavioral tests and allow differentiation between individuals with and without CAPD^(3,5,7,8)^.

The instruments mentioned in the literature are predominantly validated and standardized for children, such as the Scale of Auditory Behaviors (SAB), which effectively identifies children at risk for CAPD. This instrument is significantly correlated with the results of behavioral CAP assessment^(10)^. SAB is also effective with adults aged 18 to 35 years, showing that those with alterations in CAP had reduced scores on the scale. Furthermore, SAB was associated with the results of the Compressed Speech Test^(7)^.

The Auditory Processing Domains Questionnaire (APDQ) was developed for screening CAPD in children and adolescents aged 7 to 17 years, contributing to the distinction between attention-deficit/hyperactivity disorder and language disorders, based on reports from parents or teachers^(11)^.

In the validation phase, the instrument demonstrated high internal reliability and adequate external reliability. Factor analysis corroborated three factors aligned with the screened diagnoses, while regression models indicated a significant influence of the groups (with and without clinical alteration), age, and educational level of the caregivers on the scores. On the other hand, sex and race had no statistical significance^(11)^.

According to the authors, the instrument presented psychometric characteristics that met several criteria necessary to determine the diagnostic accuracy of tests on hearing and language, such as a sample size greater than 100, established demographic data, Pearson r values ​​(test-retest reliability) greater than 0.9, and sensitivity and specificity greater than 80%^(11)^.

Although designed for use in children, the APDQ also had satisfactory results in adults, revealing high sensitivity for the identification of CAPD in this population^(12)^.

However, questionnaires designed to assess auditory skills in Brazilian adults are still scarce. The Scale of Self-Perception of Central Auditory Processing Skills (EAPAC) was developed to address this need, consisting of 13 questions related to the auditory and academic domain, which proved capable of identifying Brazilian adults with signs of risk for alterations in auditory closure and temporal resolution skills^(13)^.

The EAPAC indicated the prevalence of auditory and academic complaints related to concentration, planning, memory, and speech comprehension in competing noise among the Brazilian adults evaluated, which can affect their performance in daily tasks^(14)^.

Although they did not define the prevalence of adults with CAPD, studies show that complaints related to speech processing under unfavorable conditions are frequent, and the negative impact on mental health and overall performance of the individual reinforces the importance of investigating the disorder in adults^(3,4,13-15)^.

Given the need to address the scarcity of instruments for Brazilian adults, expand knowledge about the EAPAC, and overcome limitations in initial studies on the scale, this study expanded the instrument, naming it EAPAC-Expanded, to encompass different complaints presented by adults that may be associated with various auditory skills. Therefore, the research aimed to validate and standardize the online application of the EAPAC-Expanded for Brazilian adults, considering CAP assessment results and the CAPD diagnosis when one skill was altered and when two or more auditory skills were altered.

## METHODS

This cross-sectional, analytical, observational study was approved by the Research Ethics Committee (COEP) of the Federal University of Minas Gerais (UFMG), under approval number 5.137.573.

### Sample

To participate in the sample, subjects had to be Brazilian adults, members of UFMG, aged 18 to 59 years, of either sex, with any education level.

The research was divided into two stages: the first consisted of the online application of the EAPAC-Expanded, with a total of 1,376 scales completed, and the second stage consisted of the in-person application of auditory and neuropsychological tests to 128 individuals.

The following exclusion criteria were applied in the second stage: obstruction of the auditory canal that prevented the performance of audiological examinations, hearing loss, regardless of type or degree, type B, C, As, or Ad tympanometry, evident syndromic, language, neurological, and/or cognitive alteration, and history of speech-language-hearing therapy for CAPD. The absence of the acoustic reflex alone was not used as an exclusion criterion for the study participants. Thus, subjects lacking the acoustic reflex at one or more frequencies, provided they did not meet the previously established exclusion criteria, remained in the analyzed sample.

### Procedures

The following assessments were carried out:

- EAPAC-Expanded^(13)^: Questions related to communicative difficulties, interpretation of verbal and nonverbal auditory stimuli, and task execution, which may be affected by impairments in auditory, cognitive, and linguistic skills, were added to broaden the scope of the EAPAC for detecting problem situations related to CAPD and to make the instrument's psychometric parameters more robust. The questions were based on research describing the symptoms of the disorder and the reported complaints. This restructured instrument, called EAPAC-Expanded, consisted of 24 questions: 15 related to the auditory domain and nine related to the academic domain. Considering the widely recognized relationship in the literature between CAPD and learning, the scale developed and validated by Abreu et al.^(13)^ was structured to encompass the various complaints that may be presented by individuals with CAPD, both in tasks involving greater auditory demand and in academic and/or professional contexts. The questions included in the originally validated instrument were retained in the expanded version. Variables related to auditory and academic/professional complaints were analyzed in combination, not alone, considering that the instrument's objective is to identify the risk for CAPD. It is important to emphasize that, given the indication of risk identified by the scale, a formal evaluation is indispensable for diagnostic confirmation and for the investigation of possible differential diagnoses. The expanded scale was submitted to analysis by three judges, speech-language-hearing pathologists with experience in the subject, who suggested the exclusion of one question because it was similar to another; hence, the instrument had 23 yes/no questions. In the EAPAC development study^(13)^, the response options were modified to a binary format after analysis by a group of expert judges. The present study maintained this response format, considering the already consolidated evaluations and recommendations. The scale was completed online, and the choice was based on the intention to assess CAP complaints in a format that overcame physical limitations, promoting greater accessibility to the assessment. Self-reported complaints received 1 point; the total score on the scale is the sum of the scored questions.
- Pure-tone audiometry, speech audiometry, and acoustic immittance: the external auditory canal was inspected using an Omni Led MD otoscope, ensuring there was no canal obstruction for the examinations. Subsequently, audiometric thresholds from 250 Hertz (Hz) to 16 Kilohertz (kHz), speech reception threshold, and speech recognition percentage were measured, also verifying the tympanometry curve and contralateral stapedial reflexes, using a Piano Inventis audiometer, TDH-49 headphones, and an AT235 Interacoustics tympanometer. The normality standards defined by the World Health Organization^(16)^ for audiometry, by Burguetti et al.^(17)^ for high-frequency audiometry, and by Jerger^(18)^ for acoustic immittance were considered. Type A tympanometry was considered when the middle ear volume was between 0.30 and 1.65 milliliters, and the peak pressure was around 0 decapascal (daPa), which could vary up to -100 daPa^(18)^. Acoustic reflexes were considered present when the threshold occurred at 70 decibels hearing level (dB HL) up to the maximum output of the device.
- CAP behavioral tests: The evaluation consisted of Speech-in-Noise test, Dichotic Digits test, Frequency Pattern test, Duration Pattern test, Random Gap Detection Test (RGDT), and Masking-Level Difference (MLD) to investigate auditory processes (low redundancy monaural speech test, dichotic listening test, temporal processing test, and binaural interaction test). Unlike the initial study on the EAPAC, the present study opted for using the RGDT test to evaluate temporal resolution, as it requires less application time than the GIN, thus allowing for a reduction in the total session duration, performed in a single session. Considering that both tests, RGDT and GIN, evaluate the same auditory skill, it was not considered that such a substitution would have a significant impact on the normative analysis of the scale. The tests used are standardized by Pereira and Schochat^(19)^ and Auditec^®(20)^ and were performed according to the application, correction, and standardization guidelines for adult tests described in the instruction manuals. Auditory processing deficit was considered when at least one auditory skill was altered, as recommended by the Brazilian Academy of Audiology^(21)^. The examinations used the Piano Inventis audiometer and the TDH-49 headphones. The selection of tests followed the national guideline, which recommends choosing at least one test for each auditory process^(21)^.
- Brief Neuropsychological Assessment Instrument – ​​NEUPSILIN^(22)^: tasks with the highest auditory demands were selected. Thus, temporospatial orientation, attention, memory, arithmetic skills, language, and executive functions (problem-solving and verbal fluency) were evaluated, following the instruction manual for application and performance classification. This instrument was used to complement the analysis stages. Its application verified the participants' cognitive skills. This approach was fundamental to ensuring that such difficulties did not negatively influence the results obtained in the assessment of auditory skills, thus contributing to the reliability and validity of the data collected in the study.

In stage 1 of the study, the UFMG’s Information Technology Directorate sent the EAPAC-Expanded, structured in Google Forms, with an informed consent form by email to 50,561 individuals from the academic community. Of these, 1,274 individuals who completed the scale were selected for stage 1, and 63 completed the scale again within a year and a half to verify auditory behavior over time.

In stage 2, the sample size calculation determined that 146 subjects should participate in the study, considering the 1,274 individuals, the 80% confidence level, and the 5% margin of error. In this phase, the assessments were carried out in one session in the simulation laboratory of UFMG’s Medical School, with rest intervals between tests.

### Statistical analysis

The data was tabulated in an Excel^®^ spreadsheet. Absolute and relative frequencies were used for qualitative variables, while quantitative data were subjected to the Shapiro-Wilk test, which rejected normality, thus using medians and quartiles.

Exploratory factor analysis verified which items would be kept in the instrument and in which domains, using the principal components method with Varimax rotation. All items with a correlation of less than 0.5 were excluded because they were not associated with any of the identified factors.

The ROC curve was applied to create cutoff points on the scale, establishing sensitivity and specificity values ​​of the instrument. The gold standard variables for comparing the scale results were the presence of CAPD with one altered test, the presence of CAPD with two or more altered tests, and test results by auditory processes.

The Wilcoxon test was used to compare the scores of the scale answered at two different time points.

All analyses considered a 5% significance level and were performed using IBM SPSS, version 25.

## RESULTS

### Sociodemographic data

Altogether, 1,376 scales were completed; considering the study participation criteria, 1,274 individuals who completed the scale were included in the research.

Most participants were female (68.4%), with a median age of 28 years, and had attended public high school (61.5%). Regarding education, individuals who were attending higher education were frequent (39.4%), followed by master’s students (22.8%). Regarding language studies, 65.6% considered themselves not fluent in any foreign language, although the majority reported studying languages ​​(82.6%).

In the second stage, 128 individuals agreed to participate in the research; after considering the exclusion criteria, the final sample consisted of 116 individuals who maintained sociodemographic characteristics similar to the sample from stage 1. The median age was 27 years, with 72.4% being female; the majority were undergraduate (44%) or master’s students (27.6%), coming from public schools (64.7%), having studied a foreign language (88%), but not being fluent (73.1%).

### Scale validation and standardization

For the validation analysis of the EAPAC-Expanded, exploratory factor analysis was applied to verify the possible grouping of variables into factors (domains), analyzing items Q1 to Q23; therefore, 23 items. These went through two rounds of grouping, following the principal components method and Varimax rotation. This showed that five items needed to be removed, as they did not correlate with the domains of the scale – i.e., their correlation was less than 0.5. After removing them, the instrument’s final version had 18 questions and five domains (Table 1).

**Table 1:** Rotated component matrix

**Question**	**Domain 1**	**Domain 2**	**Domain 3**	**Domain 4**	**Domain 5**
Q1	0.057	0.310	0.726	0.055	-0.022
Q2	0.104	0.095	0.615	0.056	0.236
Q3	0.055	0.115	0.783	0.158	0.033
Q4	-0.044	0.228	0.222	0.656	-0.023
Q5	0.160	0.671	0.206	0.070	0.071
Q7	0.115	0.537	0.191	0.106	0.051
Q8	0.026	0.779	0.176	-0.060	0.002
Q9	0.734	0.053	0.124	0.004	-0.008
Q10	0.727	0.150	0.023	-0.016	0.052
Q11	0.702	0.012	0.000	0.047	-0.003
Q12	0.732	0.095	0.083	0.136	0.004
Q15	0.093	-0.009	0.102	0.685	-0.069
Q16	0.133	0.046	-0.037	0.673	0.234
Q18	0.098	0.094	0.084	0.254	0.584
Q19	0.005	-0.088	0.129	-0.178	0.677
Q20	0.029	0.226	0.009	0.058	0.543
Q21	0.207	0.541	-0.072	0.195	0.218
Q22	0.568	0.186	0.027	0.087	0.169

The five factors together explain 50.64% of the variability of the sample. Considering that the answer "yes" is equivalent to 1 point and the answer "no" to 0 points, domain 1 has a maximum of 5 points, domain 2 has a maximum of 4 points, and domains 3, 4, and 5 have a maximum of 3 points each. Chart 1 shows the composition of the EAPAC-Expanded after the validation analysis.

**Chart 1:** EAPAC-Expanded after validation

**EAPAC – SCALE OF SELF-PERCEPTION OF CENTRAL AUDITORY PROCESSING SKILLS-EXPANDED**			
Date:_____/_____/_________			
Name:____________________________________________			
Age:____________________ Education level:_____________________________________________________			
Phone: ( ) ___________ - ____________			
Did you attend high school at a ( ) public school ( ) private school ( ) not applicable			
Are you fluent in any foreign language? ( ) yes ( ) no			
Do you study or have you studied any foreign language? If so, which one and for how long?			
**QUESTIONS**		**RESPONSES**	
		**Yes**	**No**
		**SCORE**	
		(**1**)	(0)
Q1	Do you believe you have problems detecting sound (sound in general, speech, or other sounds)?		
Q2	Do you believe you have problems locating and identifying the source of a sound (knowing where someone is calling from a distance, for example)?		
Q3	Do you believe you have trouble identifying sounds in general?		
Q4	Do you believe you have problems discriminating between sounds (differentiating speech sounds; for example, hearing S and Z)?		
Q5	Do you believe you have problems with selective and sustained attention to sound (hearing and understanding the professor's speech, even if there are other conversations in the room or external noise, for example)?		
Q6	Do you believe you have difficulty perceiving sounds in time? For example, understanding someone who speaks very quickly or who articulates words unclearly.		
Q7	Do you believe you have difficulty hearing and understanding speech in noisy situations? For example, when talking at a bus stop, in restaurants, etc.		
Q8	Do you have or have you ever had academic difficulties related to concentration at any point in your academic life or during your professional activity?		
Q9	Do you have or have you ever had academic difficulties related to memory at any point in your academic life or professional activity?		
Q10	Do you have or have you ever had academic difficulties related to planning at any point in your academic life or professional activity?		
Q11	Do you have or have you ever had academic difficulties related to learning at any point in your academic life or during your professional training?		
Q12	Do you make letter substitutions that represent similar sounds in your writing or reading? Below are some examples of letters that represent similar sounds: B – P, D – T, G – C/Q/K, V – F, Z – S, J – X/CH. E.g.: *Cola – Gola, Já – Chá, Vaca – Faca*		
Q13	Do you have difficulty making and perceiving pauses in text due to punctuation marks?		
Q14	Do you have difficulty recognizing when someone is trying to convey a different meaning by changing their tone of voice?		
Q15	Do you have difficulty understanding jokes or words with double meanings?		
Q16	Do you have difficulty perceiving and reproducing rhythms?		
Q17	If you are talking to someone and you miss a part of what they said, do you have difficulty understanding their entire message/speech?		
Q18	Do you have difficulty completing complex tasks (that require formulating and giving answers) within the stipulated time?		

**Figure 1:**
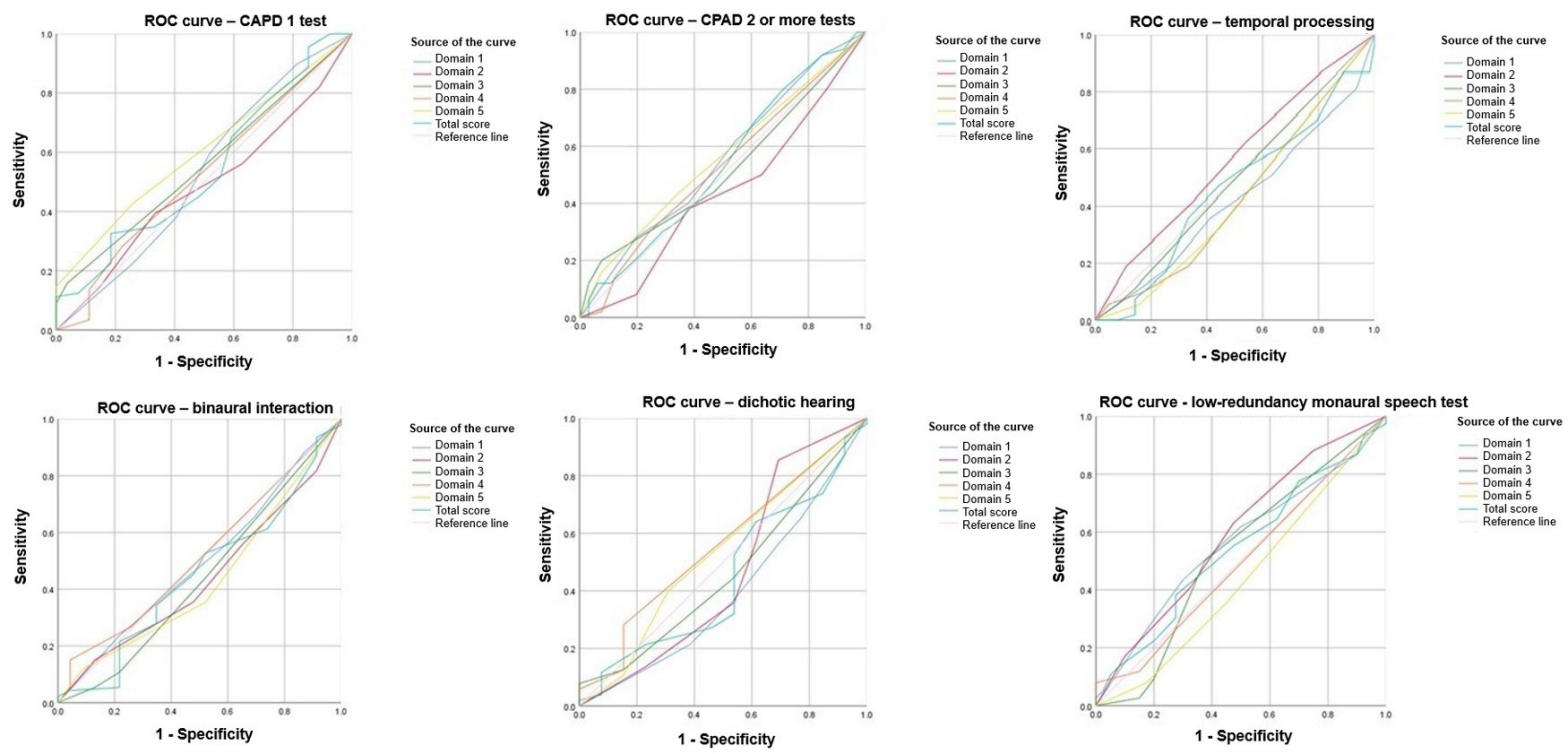
ROC curves: diagnosis of CAPD by amount of abnormal test and auditory processes

After validating the instrument, the sensitivity and specificity values ​​were analyzed, and the cutoff was defined considering the presence of CAPD when one auditory skill was altered (CAPD 1 test) and when there was an alteration in two or more auditory skills (CAPD 2 or more tests), considering the CAP assessment results by auditory processes, the scores per domain, and the total scale score. However, isolated questions were not considered. The data are presented in Table 2 and the ROC curves in Figure 1.

**Table 2:** EAPAC-Expanded normative data

**Gold standard variables**	**EAPAC-Expanded**		**Area**	**p-value**	**95% CI**		**Cutoff**	**Sensitivity**	**Specificity**
**CAPD 1 test**	**Domain1**		0.528	0.664	0.394	0.661	2.5	59.6%	48.1%
	**Domain2**		0.483	0.784	0.363	0.602	2.5	39.3%	66.7%
	**Domain3**		0.557	0.369	0.440	0.674	0.5	47.2%	59.3%
	**Domain4**		0.523	0.717	0.398	0.648	0.5	28.1%	77.8%
	**Domain5**		0.603	0.107	0.490	0.715	0.5	42.7%	74.1%
	**Total scale**		0.542	0.509	0.417	0.667	4.5	65.2%	40.7%
**CAPD 2 or more tests**	**Domain1**		0.552	0.342	0.446	0.657	2.5	62.0%	45.5%
	**Domain2**		0.433	0.221	0.328	0.539	2.5	38.0%	62.1%
	**Domain3**		0.517	0.755	0.408	0.626	0.5	44.0%	53.0%
	**Domain4**		0.523	0.666	0.417	0.630	0.5	30.0%	75.8%
	**Domain5**		0.557	0.295	0.450	0.664	0.5	44.0%	65.2%
	**Total scale**		0.534	0.531	0.429	0.640	5.5	54.0%	48.5%
**Temporal processing**		Domain1	0.420	0.138	0.315	0.525	2.5	50.9%	36.5%
		Domain2	0.560	0.265	0.455	0.665	1.5	62.3%	46.0%
		Domain3	0.490	0.846	0.384	0.595	0.5	45.3%	54.0%
		Domain4	0.431	0.199	0.326	0.535	0.5	18.9%	66.7%
		Domain5	0.425	0.167	0.321	0.529	0.5	32.1%	55.6%
		Total scale	0.456	0.418	0.350	0.562	6.5	47.2%	55.6%
**Binaural interaction**		Domain1	0.494	0.926	0.363	0.624	2.5	57.0%	39.1%
		Domain2	0.436	0.341	0.311	0.560	2.5	35.5%	52.2%
		Domain3	0.439	0.363	0.301	0.576	0.5	44.1%	47.8%
		Domain4	0.516	0.817	0.388	0.644	0.5	26.9%	73.9%
		Domain5	0.432	0.314	0.303	0.561	0.5	35.5%	47.8%
		Total scale	0.450	0.463	0.319	0.582	5.5	52.7%	57.8%
**Dichotic hearing**		Domain1	0.394	0.212	0.227	0.561	2.5	56.3%	30.8%
		Domain2	0.468	0.707	0.271	0.665	2.5	35.9%	46.2%
		Domain3	0.460	0.636	0.296	0.623	0.5	44.7%	46.2%
		Domain4	0.556	0.509	0.395	0.717	0.5	28.2%	84.6%
		Domain5	0.531	0.716	0.360	0.702	0.5	39.8%	69.2%
		Total scale	0.446	0.526	0.279	0.613	4.5	64.1%	38.5%
**Low-redundancy monaural speech test**		Domain1	0.560	0.288	0.452	0.668	2.5	61.8%	50.0%
		Domain2	0.595	0.094	0.484	0.705	1.5	63.2%	52.5%
		Domain3	0.525	0.653	0.409	0.642	0.5	50.0%	62.5%
		Domain4	0.497	0.951	0.386	0.607	0.5	26.3%	72.5%
		Domain5	0.439	0.284	0.327	0.552	0.5	35.5%	50.0%
		Total scale	0.543	0.450	0.434	0.652	3.5	77.6%	70.0%

**Caption:** CI = confidence interval, p-value ≤ 0.05

**Caption:** CAPD = central auditory processing disorder

The median of the total scale score was 6 points, the first quartile was 3 points, and the third quartile was 8 points. The minimum score found was 0, and the maximum was 15 points.

A comparison was made between the scores (total and by domain) of 63 individuals who responded to the EAPAC-Expanded at two time points, using the Wilcoxon test, finding no significant difference between the test and retest responses (Table 3).

**Table 3:** Comparison of EAPAC-Expanded results at two different time points

	**Time point 1**	**Time point 2**	**p-value** ^*^
**Domain 1**	3 (1 – 4)	2 (1 – 4)	0.713
**Domain 2**	2 (1 – 3)	2 (1 – 3)	0.464
**Domain 3**	0 (0 – 1)	0 (0 – 1)	0.928
**Domain 4**	0 (0 – 1)	0 (0 – 0)	0.207
**Domain 5**	0 (0 – 1)	1 (0 – 1)	0.487
**Total scale**	6 (3 – 9)	6 (4 – 9)	0.984

* Wilcoxon test. Data presented as median and quartiles

### Self-reported complaints and cognitive and audiological assessment

The most frequent self-reported complaints were academic difficulty related to concentration (63.7%), planning (61.8%), and memory (60.3%), difficulty hearing and understanding speech in noisy environments (58.6%), perceiving sound over time (48.6%), and attention to sound (47.1%).

Regarding the results of the audiological evaluation, 86.2% of individuals had contralateral stapedial reflexes in three or four of the frequencies evaluated in the right and left ears. The median of the audiometric thresholds from 0.5 to 4 kHz was equal to 10 dB HL bilaterally, and the minimum and maximum values ​​were equal to 0 and 20 dB HL bilaterally. The median audiometric thresholds from 9 to 16 kHz were 8.3 dB HL in the right ear and 9.1 dB HL in the left ear, with minimum and maximum values ​​of 2.5 and 20 dB HL in the right ear and 2.5 and 21 dB HL in the left ear.

Regarding the neuropsychological assessment, most of the sample performed adequately in cognitive skills. Therefore, no individual had significant alterations in various skills that would compromise the assessments. Hence, no participant needed to be excluded based on these results.

Those who presented a diagnosis of psychiatric and/or neurological disorder were excluded during sample selection.

Regarding the frequency of CAPD diagnosis, 76.7% of the sample presented the disorder when at least one auditory skill was altered in the assessment. Considering the occurrence of alteration in CAP when two or more tests presented results below the reference values, 43.1% of individuals received a CAPD diagnosis.

Regarding the CAP assessment results by auditory processes, 52.6% obtained normal results for the temporal processing tests, 80.2% in the binaural interaction test, 88.8% in the dichotic listening test, and 66.4% in the low-redundancy monaural speech test. Thus, the temporal processing and low-redundancy monaural speech tests showed a higher frequency of altered results.

## DISCUSSION

CAPD can occur in normal hearing adults, hindering communication and performance in various areas of life^(1-3,5)^. Therefore, it is necessary to investigate CAPD in this population, and questionnaires point to the daily difficulties faced^(5,7,23)^. Due to the lack of instruments developed for Brazilian adults^(24)^, this study aimed to validate and standardize the EAPAC-Expanded.

The validation process followed the criteria recommended in the literature, such as content validity, verified through expert analysis, criterion validity, through comparison of the EAPAC-Expanded data with the measures considered the gold standard, and construct validity, through factor analysis^(25)^.

Exploratory factor analysis was used to verify the patterns of all data, thus identifying the correlation between the variables of the instrument. The investigation led to the exclusion of five questions with low factor loadings (i.e., less than 0.5), which means that they did not converge to a common point, not showing a correlation. The remaining questions showed correlations with values ​​close to or higher than those established in the literature (equal to or greater than 0.7)^(25)^.

After verifying convergent validity, five domains were identified in the scale, unlike the non-expanded version, which has only two: auditory and academic^(13)^. Therefore, the questions added to the instrument made it possible to identify several auditory and cognitive skills investigated by the scale and to group the questions based on this data.

The domains were defined based on questions about academic performance (domain 1), attention and speech comprehension under unfavorable conditions (domain 2), sound perception, localization, and identification (domain 3), sound discrimination and gap identification (domain 4), and intonation and rhythm (domain 5). Together, these domains explained 50.64% of the sample variability.

The variability, related to the influence of the characteristics and size of the sample on the factors analyzed, presented results equivalent to those obtained in the psychometric evaluation of the APDQ instrument, whose values ​​were considered satisfactory. In the aforementioned study, the questionnaire demonstrated a variance of 50.3% when considering its three domains^(11)^.

The excluded items were related to short-term memory and linguistic skills. The analysis of items that correlated with the domains showed the persistence of questions about learning difficulties and with greater involvement of auditory skills^(26)^.

After validating the instrument, ROC curves were created to analyze the sensitivity, specificity, and cutoff of the instrument. The statistical analysis for scale standardization considered multiple variables. Thus, the following were used as gold standards for the construction of the ROC curve: the diagnosis of CAPD with alteration in a behavioral test (i.e., presence of an altered skill), occurrence of two or more altered tests, and the result of the evaluation of each auditory process individually. In total, six variables were considered, whose data were presented separately.

The area under the curve is expected to have a minimum value of 70% and a significant p-value. However, the areas under the curves were below the reference and without statistical significance, indicating low sensitivity and specificity and weak cutoffs.

Considering the presence of CAPD due to an alteration in a CAP behavioral test, the scale showed a sensitivity of 65.2% and a specificity of 40.7%, with a cutoff of 4.5. With two or more altered CAP tests, the instrument identified 54% of individuals with CAPD and 48.5% of those without the disorder, with a minimum score of 5.5.

Analysis of the scale's properties by auditory processes showed sensitivity and specificity equal to 47.2% and 55.6%, respectively, for temporal processing; 52.7% and 57.8% for the binaural interaction test; 64.1% and 38.5% for the dichotic listening assessment, and 77.6% and 70% for the low-redundancy monaural speech test. Cutoffs were 6.5 points for alteration in temporal processing, 5.5 points for alteration in the binaural interaction test, 4.5 points for alteration in the dichotic listening test, and 3.5 points for alteration in the low-redundancy monaural speech test.

The initial study of the EAPAC showed scores of 5 and 6 to indicate alteration in the SIN and GIN tests, respectively. Sensitivity and specificity were 74% and 56% for the SIN test, and 62% and 51% for the GIN test^(13)^. Compared with the EAPAC-Expanded, it was found that the values ​​differed little in relation to the psychometric measures of the EAPAC.

The scores of the APDQ domains – Auditory Processing, Attention Deficit, and Language Disorder – allowed the correct classification of more than 80% of the study participants (no clinical alteration, CAP alteration, ADHD, and language disorder), according to the recommendations of specialized literature. Most of the identified cutoffs ​​showed sensitivity and specificity greater than 80%^(11)^. In addition, previous evaluation of this instrument in adults indicated high sensitivity, reaching 85.7%^(12)^. The comparison of results obtained through the instrument with those from the EAPAC-Expanded analysis showed a difference between the two: EAPAC-Expanded presented values ​​below what is recommended by the literature.

Although the psychometric measures of the EAPAC-Expanded are not very robust for identifying the risk for CAPD, the scale proved useful for analyzing the hearing difficulties presented in the naturalistic environment (i.e., the impact on the subject's life), assisting evaluators in understanding the case, developing strategies to minimize the difficulties, and monitoring clinical evolution.

Self-reported difficulties scored 1 point on the scale; thus, the higher the score, the more self-reported complaints. The sample presented a median score equal to 6 points, corroborating the study by Abreu et al.^(13)^, which found an average score of 6.1.

When comparing auditory behavior over time, no significant changes were observed. Changes in the auditory nerve pathway result from advancing age, due to neuronal losses that begin between 35 and 40 years of age. They cause auditory complaints, such as altered speech perception^(3)^, which was not identified in the sample. There was no change in the frequency of self-reported complaints, as the median age of the sample is lower than the age prone to these losses.

The EAPAC-Expanded identified that the most frequently indicated items (i.e., the most prevalent complaints) were learning difficulties due to concentration, planning, and memory, and difficulty understanding speech in noise, perceiving sound in time, and maintaining attention to sound.

The learning process is influenced by cognitive skills, such as attention, memory, and executive functions, which help to organize and integrate information, leading to behavioral change and the understanding of new concepts. With school time, tasks become more complex, requiring more cognitive input. Deficits in these skills, especially attention, memory, and planning, which are also essential for CAP, can cause learning problems^(27)^, as evidenced in the research.

These cognitive skills are important for CAP, as they direct focus to the auditory stimulus, inhibit distractors, and promote the storage and interpretation of information to generate correct responses to the auditory stimulus^(2,4)^.

According to the literature, individuals with impairments in memory, executive function, and attention have difficulty identifying speech in noisy environments^(2)^, which, added to alterations in auditory skills, cause complaints related to sound attention and identification^(7,28,29)^.

Studies frequently report complaints related to difficulty understanding sound in noise. In the research by Cancel et al.^(6)^, 44% of normal hearing adults with a median age of 35 years reported a feeling of hearing loss or hearing difficulty in noise. The study by Pereira et al.^(14)^ also identified a high percentage of auditory complaints related to speech comprehension in noise (56.8%).

Studies with children and adolescents also identify such symptoms. In the study by O’Hara and Mealings^(11)^, the APDQ showed that individuals with CAPD have significant difficulties understanding speech in noisy environments, listening while multitasking, and listening to speakers with unclear articulation. Participants diagnosed with ADHD demonstrated challenges mainly related to attention, auditory memory, and greater susceptibility to distractions caused by ambient noise. The symptoms mapped by the APDQ revealed similarity to the difficulties indicated by the EAPAC-Expanded.

Perceiving, identifying, and recognizing sound demands common auditory and cognitive skills, which justifies the frequent self-reported auditory difficulties in this research. Furthermore, the results were consistent with the data in the literature.

The frequency of CAPD in the sample was high, both when a single behavioral test was altered and when two or more showed alteration. The prevalence of CAPD is not well defined, but the literature suggests an occurrence between 0.5% and 1%, increasing when it occurs with other factors^(30)^.

The results of this study corroborate the literature^(6)^, which also shows a high percentage of CAPD. According to the authors, 51% of the sample presented CAPD. The high prevalence in the study on the EAPAC-Expanded may be because most subjects who expressed a desire to participate in the research had auditory complaints, being, therefore, suggestive of CAPD, which may have influenced the results of this research. Hence, further studies with subjects without CAPD are necessary.

According to the Brazilian Academy of Audiology^(21)^, a single altered behavioral test is sufficient for the diagnosis of CAPD, which may increase the prevalence of the disorder. Considering the occurrence of CAPD in the general population, the interference of cognitive and linguistic factors in auditory performance, and the inadequate diagnosis of the disorder^(30)^, it is important to discuss and define the criteria for the diagnosis of CAPD.

Regarding the CAP assessment, most tests showed normal results, but temporal auditory processing had more alterations, followed by the low-redundancy monaural speech test. The data partially agreed with the literature, which pointed to deficits in dichotic listening (binaural integration) and in the low-redundancy monaural speech test (speech in noise) among adults referred for CAP evaluation due to hearing complaints^(6)^.

The perception of variations in sound as a function of time involves temporal ordering and resolution, which make up temporal auditory processing. These skills help to understand speech and recognize phonemes and variations in the suprasegmental aspects of speech – i.e., to perceive and identify the characteristics of sound over time^(31,32)^.

The literature has shown that young men detect gaps better than women^(31)^, and those with CAPD showed worse results in auditory temporal ordering and resolution tests^(31)^. Therefore, the high percentage of alteration in temporal auditory processing in the present research can be justified by the majority of women and the high percentage of CAPD in the sample.

Speech is more easily understood when presented in good sound conditions and combined with extrinsic factors, such as context and familiarity with words, the theme of the discourse, and the rules of the language. However, under poor listening conditions, speech comprehension becomes a challenging situation, requiring greater use of cognitive and auditory resources (intrinsic resources) for speech recognition^(31)^.

Listening to monosyllabic words in noise was difficult for the study sample. Difficulty understanding speech in noise was one of the common self-reported complaints on the expanded scale and was identified in the behavioral test. Thus, short words, and therefore with few auditory cues and low redundancy, were difficult to recognize, even if they were familiar words^(31,33)^. The study by Turcatto et al.^(7)^ also showed that adults with and without CAPD had difficulty with the low-redundancy monaural speech task with monosyllabic words.

This study contributed to investigations on the EAPAC-Expanded, highlighting its usefulness in understanding the impact of difficulties in analyzing auditory stimuli in everyday tasks in adults, given the lack of adequate tools. However, the research had limitations, such as the lack of investigation into the influence of tasks that enhance auditory performance, such as musical training, on the subjects' responses, and a smaller sample size than desired. Another relevant aspect for analysis refers to ascertaining the influence of issues associated with learning, which may be linked to non-auditory or cognitive components, on the results obtained in the validation of the instrument.

Future studies should consider the inclusion of a control group of individuals with auditory and cognitive performance within normal parameters. It is also important that the EAPAC-Expanded be directly applied by speech-language-hearing pathologists, instead of being self-administered by the participant without professional supervision, in order to adequately compare the results obtained in the two application modalities.

## CONCLUSION

The EAPAC-Expanded did not present adequate psychometric parameters in the validation and standardization processes for identifying individuals at risk for CAPD. Nevertheless, it proved to be useful for investigating people’s difficulties in a naturalistic environment. The instrument is based on self-reported complaints; therefore. it is relevant to point out the impact of the problem on the subject's life. The scale is thus able to guide the evaluator in defining strategies that can minimize self-reported complaints and in understanding the impact of the reported difficulties. Thus, the EAPAC-Expanded can complement the CAP assessment battery as an instrument that identifies the individual's auditory self-perception.
