# Supplementary material for: Validation and standardization of the Scale of Self-Perception of Central Auditory Processing Skills (EAPAC) - expanded
Source: Codas. 2026 Jun 19;38(4):e20250215. doi: 10.1590/2317-1782/e20250215en (PMC13322490; doi:10.1590/2317-1782/e20250215en)
Supplement: Translation

# Translation: Validação e normatização da Escala de Autopercepção de Habilidades do Processamento Auditivo Central (EAPAC) - expandida

**DOI:** 10.1590/2317-1782/e20250215pt

**Authors:** Luciana Cássia de Jesus, Luciana Mendonça Alves, Luciana Macedo de Resende

**Affiliations:** 1 Programa de Pós-graduação em Ciências Fonoaudiológicas, Faculdade de Medicina, Universidade Federal de Minas Gerais – UFMG - Belo Horizonte (MG), Brasil.; 2 Departamento de Fonoaudiologia, Faculdade de Medicina, Universidade Federal de Minas Gerais – UFMG - Belo Horizonte (MG), Brasil.

**Keywords:** Audição, Percepção Auditiva, Inquéritos e Questionários, Autorrelato, Estudo de Validação, Cognição, Comunicação, Adulto

## Abstract

**Objetivo:**

validar e normatizar a aplicação on-line da Escala de Autopercepção de Habilidades do Processamento Auditivo Central (EAPAC) – expandida para adultos brasileiros.

**Método:**

A Escala de Autopercepção de Habilidades do Processamento Auditivo Central foi expandida e respondida por 1274 indivíduos brasileiros com idade entre 18 e 59 anos. Desses, 116 sujeitos foram submetidos à avaliação neuropsicológica e auditiva e 63 foram selecionados para responder à escala em um segundo momento. Foram excluídos aqueles com perda auditiva de qualquer tipo e grau, histórico de terapia fonoaudiológica para o transtorno do processamento auditivo central e alteração psiquiátrica e/ou neurológica. Foi realizada a análise descritiva das variáveis, a análise fatorial exploratória e a curva ROC, para validação e normatização. O teste de Wilcoxon foi utilizado para comparar as respostas dos sujeitos que responderam à escala em dois momentos.

**Resultados:**

Após a análise fatorial exploratória, cinco questões foram excluídas, pois não correlacionaram com os domínios da escala. As curvas ROC, construídas considerando a presença do transtorno do processamento auditivo e o resultado por processos auditivos, não apontaram valores significativos, sendo assim, a escala apresentou valores frágeis de sensibilidade e especificidade. Não houve diferença significativa entre as respostas dos sujeitos, na escala, em dois momentos diferentes.

**Conclusão:**

A escala expandida não apresentou padrões psicométricos adequados para identificação do risco para o transtorno do processamento auditivo central, mas mostrou-se útil para investigar as queixas auditivas em tarefas do cotidiano, e, assim, auxiliar na seleção de estratégias para minimizar o impacto negativo das dificuldades referidas.

## INTRODUÇÃO

A audição é crucial para a execução de tarefas do cotidiano, profissionais, acadêmicas e comunicativas, permitindo a captação e análise do som para gerar respostas adequadas^(1)^. O Processamento Auditivo Central (PAC) é responsável por discriminar e interpretar essas mensagens sonoras, apoiado por habilidades cognitivas, como a memória operacional, a atenção e a velocidade do processamento da informação^(1-4)^.

O transtorno do processamento auditivo central (TPAC) ocorre na presença da audição normal, prejudicando a análise sonora, especialmente em ambientes acusticamente desafiadores^(1-3)^. Embora afetem principalmente crianças, adultos também mostram sintomas, impactando sua comunicação e o desempenho social, o que torna relevante estudar as habilidades auditivas nesse grupo^(1-5)^.

Um estudo demonstrou que muitos adultos com queixas auditivas e limiares audiométricos normais não são encaminhados para a avaliação do PAC. A maioria autorreferiu problemas auditivos moderados a graves no *Hearing Handicap Inventory for Adults* – HHIA-S (Inventário de Handicap Auditivo para Adultos). Entre os indivíduos que queixaram de dificuldade auditiva, mais da metade apresentou TPAC^(6)^.

A avaliação do PAC é realizada por meio de testes comportamentais, acrescido da avaliação eletrofisiológica. É recomendado usar questionários de autopercepção para identificar o risco para o TPAC, analisar a percepção do sujeito sobre seu estado de saúde, registrar as dificuldades apresentadas e elaborar estratégias terapêuticas, visto que são recursos simples e de baixo custo^(3,5,7-9)^.

Os checklists de autopercepção auditiva correlacionam com as habilidades auditivas avaliadas por meio dos testes comportamentais e permitem diferenciar indivíduos com e sem o TPAC^(3,5,7,8)^.

Os instrumentos mencionados na literatura são predominantemente validados e normatizados para o público infantil, como a *Scale of Auditory Behaviors* (SAB), que demonstra eficácia para identificar crianças com risco para Transtorno do Processamento Auditivo Central (TPAC). O referido instrumento apresenta correlações significativas com os resultados da avaliação comportamental do PAC^(10)^. Quando aplicada em adultos entre 18 e 35 anos*, a SAB ta*mbém demonstrou eficácia, evidenciando que aqueles com alterações no PAC apresentaram pontuação reduzida na escala. Ademais, a SAB mostrou associação com os resultados do Teste de Fala Comprimida^(7)^.

O *Auditory Processing Domains Questionnaire* (APDQ) foi desenvolvido para o rastreamento de TPAC em crianças e adolescentes de 7 a 17 anos, além de contribuir para a distinção entre Transtorno de Déficit de Atenção e Hiperatividade e distúrbios de linguagem, com base nos relatos de pais ou professores^(11)^.

Na etapa de validação, o instrumento demonstrou alta confiabilidade interna e adequada confiabilidade externa. A análise fatorial corroborou três fatores alinhados aos diagnósticos rastreados, enquanto os modelos de regressão indicaram influência significativa dos grupos (com e sem alteração clínica), idade e nível de escolaridade dos responsáveis nos escores obtidos; por outro lado, gênero e raça não apresentaram significância estatística^(11)^.

Segundo os autores, o instrumento apresentou características psicométricas que atenderam vários critérios necessários para determinar a precisão diagnóstica de testes sobre a audição e linguagem, como tamanho da amostra superior a 100, dados demográficos estabelecidos, valores Pearson *r* (confiabilidade teste-reteste) superior a 0,9, sensibilidade e especificidade maiores que 80%^(11)^.

Embora tenha sido projetado para uso infantil, o APDQ também mostrou resultados satisfatórios em adultos, revelando sensibilidade elevada para a identificação de TPAC nesta população^(12)^.

No entanto, ainda são escassos os questionários destinados à avaliação das habilidades auditivas em adultos brasileiros. Para suprir essa necessidade, foi elaborada a Escala de Autopercepção de Habilidades do Processamento Auditivo Central (EAPAC), composta por 13 questões relacionadas ao domínio auditivo e acadêmico, que se mostrou capaz de identificar adultos brasileiros com sinais de risco para a alteração nas habilidades de fechamento auditivo e resolução temporal^(13)^.

A EAPAC apontou que, para os adultos brasileiros avaliados, foram prevalentes as queixas auditivas e acadêmicas relacionadas à concentração, planejamento e memória e compreensão da fala na presença de ruído competitivo, o que pode afetar o desempenho desses indivíduos nas tarefas do cotidiano^(14)^.

Apesar de não terem definido a prevalência de adultos com TPAC, os estudos mostram ser frequente a queixa relacionada ao processamento da fala em condições desfavoráveis e o reflexo negativo na saúde mental e no desempenho geral do indivíduo, o que reforça a importância de investigar a presença do transtorno em adultos^(3,4,13-15)^.

Diante da necessidade de suprir a escassez de instrumentos direcionados à população adulta brasileira, ampliar os conhecimentos sobre a EAPAC e suprir as limitações nos estudos iniciais sobre a escala, o presente estudo ampliou o instrumento, intitulando-o EAPAC – expandida, para abranger diferentes queixas apresentadas pelo adulto, que podem ser associadas às diversas habilidades auditivas. Portanto, a pesquisa teve o objetivo de validar e normatizar a aplicação on-line da Escala de Autopercepção de Habilidades do Processamento Auditivo Central (EAPAC) – expandida, para adultos brasileiros considerando os resultados da avaliação do processamento auditivo central, o diagnóstico do TPAC quando uma habilidade estava alterada e quando duas ou mais habilidades auditivas mostravam-se alteradas.

## MÉTODO

O estudo observacional analítico transversal foi aprovado pelo Comitê de Ética em Pesquisa (COEP) da Universidade Federal de Minas Gerais (UFMG), sob parecer n.º 5.137.573.

### Amostra

Para participar da amostra, os sujeitos deveriam ser adultos brasileiros, membros da UFMG, com idade entre 18 e 59 anos, de ambos os sexos e de qualquer escolaridade.

A pesquisa foi dividida em duas etapas: a primeira consistiu na aplicação on-line da EAPAC – expandida, com o total de 1376 escalas preenchidas, e a segunda etapa na aplicação presencial dos testes auditivos e neuropsicológico em 128 indivíduos.

Os critérios de exclusão foram aplicados na segunda etapa e compreendeu: obstrução do conduto auditivo que impedisse a realização dos exames audiológicos, perda auditiva, independentemente do tipo ou grau, curva timpanométrica do tipo B, C, Ar e Ad, alteração sindrômica, de linguagem, neurológica e/ou cognitiva evidente, e histórico de terapia fonoaudiológica para o TPAC. A ausência do reflexo acústico, isoladamente, não foi utilizada como critério de exclusão para os participantes do estudo. Assim, sujeitos que apresentaram ausência do reflexo acústico em uma ou mais frequências, desde que não preenchessem os critérios de exclusão estipulados previamente, permaneceram incluídos na amostra analisada.

### Procedimentos

Foram realizadas as seguintes avaliações:

- Escala de Autopercepção de Habilidades do Processamento Auditivo Central (EAPAC) – expandida^(13)^: com o intuito de ampliar a abrangência da EAPAC para a detecção de situações problemas relacionados ao TPAC e tornar os parâmetros psicométricos do instrumento mais robustos, foram acrescidas questões relacionadas às dificuldades comunicativas, à interpretação do estímulo sonoro, verbal ou não, e à execução de tarefas, que podem ser afetadas por prejuízos nas habilidades auditivas, cognitivas e linguísticas. As questões foram baseadas em pesquisas que descrevem os sintomas do transtorno e as queixas relatadas. Este instrumento reestruturado, denominado EAPAC – expandida, foi composto por 24 questões, sendo 15 perguntas relacionadas ao domínio auditivo e nove perguntas relacionadas ao domínio acadêmico. Considerando a relação amplamente reconhecida na literatura entre o PAC e a aprendizagem, a escala desenvolvida e validada por Abreu e colaboradores^(13)^ foi estruturada de modo a contemplar as diversas queixas que podem ser apresentadas por indivíduos com TPAC, tanto em tarefas que envolvem maior demanda auditiva quanto em contextos acadêmicos e/ou profissionais. As questões integrantes do instrumento originalmente validado foram mantidas na versão expandida. As variáveis relacionadas às queixas auditivas e acadêmicas/profissionais foram analisadas de forma integrada, e não de maneira isolada, considerando que o objetivo do instrumento é identificar o risco para o TPAC. Ressalta-se que, diante da indicação de risco identificada pela escala, é indispensável a realização de avaliação formal para confirmação diagnóstica, bem como para a investigação de possíveis diagnósticos diferenciais. A escala expandida foi submetida à análise de três juízes, fonoaudiólogas com experiência sobre o tema, que sugeriram a exclusão de uma questão, pois era semelhante à outra, e o instrumento passou a ter 23 perguntas, com opção de respostas (sim ou não). No estudo de desenvolvimento da EAPAC^(13)^, as opções de resposta foram modificadas para um formato binário, após análise realizada por um grupo de juízes especialistas. No presente estudo, optou-se pela manutenção desse formato de resposta, considerando as avaliações e recomendações já consolidadas. A escala foi respondida no ambiente virtual e a escolha foi fundamentada na intenção de avaliar as queixas relacionadas ao PAC em um formato que ultrapassasse as limitações físicas, promovendo maior acessibilidade à avaliação. As queixas autorreferidas receberam um ponto e o escore total da escala é a soma das questões pontuadas.
- Audiometria tonal limiar, vocal e imitanciometria: foi realizada a inspeção do conduto auditivo externo com o uso do otoscópio marca Omni Led MD, sendo necessário que não houvesse obstrução do conduto para a realização dos exames. Em seguida, foi realizada a pesquisa dos limiares audiométrico de 250 *Hertz* (Hz) a 16 *Quilohertz* (kHz), do limiar de recepção de fala e do percentual de reconhecimento de fala, a verificação da curva timpanométrica e dos reflexos estapedianos contralaterais, por meio do audiômetro marca *Piano Inventis*, fone TDH-49 e do imitanciômetro marca AT235 *Interacoustics*. Foram considerados os padrões de normalidade definidos pela Organização Mundial da Saúde^(16)^ para a audiometria, por Burguetti e colaboradores^(17)^ para a audiometria de alta frequência e por Jerger^(18)^ para a imitanciometria. Para a timpanometria, foi considerada a curva A quando o volume da orelha média estava compreendido entre 0,30 e 1,65 mililitro e a pressão do pico em torno de 0 decapascal (daPa), podendo variar até -100daPa^(18)^. Já os reflexos acústicos foram considerados presentes quando o limiar ocorria em intensidades a partir de 70 decibel de nível de audibilidade (dbNA) até a saída máxima do aparelho.
- Testes comportamentais do PAC – a avaliação foi composta por: teste Fala com Ruído, Dicótico de Dígitos, Padrão de Frequência, Padrão de Duração, *Random Gap Detection Test* (RGDT) e *Masking-Level Difference* (MLD), para investigação dos processos auditivos (teste de fala monoaural de baixa redundância, de escuta dicótica, processamento temporal e de interação binaural). Diferentemente do estudo inicial sobre a EAPAC, o presente estudo optou pela utilização do teste RGDT para a avaliação da resolução temporal, por demandar menor tempo de aplicação em comparação ao GIN, possibilitando, assim, a redução da duração total da sessão, realizada em um único momento. Considerando que ambos os testes, RGDT e GIN, avaliam a mesma habilidade auditiva, não se considerou que tal substituição acarretaria impacto significativo na análise normativa da escala. Os testes utilizados são padronizados por Pereira e Schochat^(19)^ e Auditec®^(20)^ e foram realizados conforme a orientação de aplicação, correção e padronização dos testes para adultos, descritos nos manuais de instrução. Foi considerado o déficit no processamento auditivo quando pelo menos uma habilidade auditiva apresentava alteração, conforme preconiza a Academia Brasileira de Audiologia^(21)^. Para a realização dos exames, foi utilizado o audiômetro marca *Piano Inventis* e o fone TDH-49. A seleção dos testes seguiu a orientação da diretriz nacional, que recomenda a escolha de pelo menos um teste para cada processo auditivo^(21)^.
- Instrumento de Avaliação Neuropsicológica Breve – NEUPSILIN^(22)^: foram selecionadas as tarefas com maior demanda auditiva. Assim, foram avaliadas a orientação têmporo-espacial, atenção, memória, habilidades aritméticas, linguagem e funções executivas (resolução de problemas e fluência verbal), seguindo as orientações do manual de instrução para a aplicação e classificação do desempenho. O instrumento em questão foi empregado para complementar as etapas da análise. A aplicação permitiu verificar as habilidades cognitivas dos participantes. Essa abordagem foi fundamental para assegurar que tais dificuldades não influenciassem de modo negativo os resultados obtidos na avaliação das habilidades auditivas, contribuindo assim para a fidedignidade e validade dos dados coletados no estudo.

Na etapa 1 do estudo, a Diretoria de Tecnologia da Informação da UFMG enviou a EAPAC – expandida, estruturada no Google *Forms*, com o Termo de Consentimento Livre Esclarecido (TCLE) por correio eletrônico, para 50.561 indivíduos da comunidade acadêmica. Desses, 1274 indivíduos que preencheram a escala foram selecionados para a etapa 1, sendo que 63 preencheram novamente a escala no intervalo de um ano e meio para verificar o comportamento auditivo ao longo do tempo.

Na etapa 2, o cálculo amostral definiu que 146 sujeitos deveriam participar do estudo, considerando os 1274 indivíduos, o grau de confiança de 80% e a margem de erro de 5%. Nessa fase, as avaliações foram realizadas no laboratório de simulação da Faculdade de Medicina da UFMG, em uma sessão, com intervalos de descanso entre os testes.

### Análise estatística

Foi realizada a tabulação dos dados na planilha no Excel®. Para as variáveis qualitativas, foram utilizadas as frequências absolutas e relativas. Os dados quantitativos foram submetidos ao teste de *Shapiro-Wilk*, que rejeitou a normalidade. Assim, foram utilizadas as medianas e os quartis.

A análise fatorial exploratória verificou quais itens seriam mantidos no instrumento e em quais domínios. Utilizou-se o método de componentes principais com rotação *Varimax*. Todos os itens com correlação inferior a 0,5 foram excluídos por não se associarem a nenhum dos fatores identificados.

Para criação de pontos de corte na escala, foi aplicada a curva ROC. Foram estabelecidos os valores de sensibilidade e especificidade do instrumento. As variáveis padrão-ouro para comparar os resultados da escala foram: presença de TPAC com 1 teste alterado, presença de TPAC com 2 ou mais testes alterados e resultado dos testes por processos auditivos.

Para a comparação entre as pontuações da escala respondida em dois momentos diferentes, foi utilizado o teste *Wilcoxon*.

Todas as análises consideraram nível de significância de 5% e foram realizadas no software IBM SPSS versão 25.

## RESULTADOS

### Dados sociodemográficos

Foram respondidas 1376 escalas e, considerando os critérios para participação do estudo, 1274 indivíduos, que preencheram a escala, foram incluídos na pesquisa.

A maioria dos participantes era do gênero feminino (68,4%), com idade mediana de 28 anos e tinham cursado o ensino médio em escola pública (61,5%). Quanto à escolaridade, foram frequentes os indivíduos que cursavam o ensino superior (39,4%), seguido de pós-graduação *stricto sensu* (22,8%). Sobre o estudo de língua, 65,6% consideraram não ter fluência em alguma língua estrangeira, apesar da maioria relatar o estudo de línguas (82,6%).

Na segunda etapa, 128 indivíduos aceitaram participar da pesquisa e, após considerar os critérios de exclusão, a amostra final foi composta por 116 indivíduos que mantiveram características sociodemográficas semelhantes à amostra da etapa 1. A idade mediana foi igual a 27 anos, sendo 72,4% do gênero feminino, a maioria com ensino superior incompleto (44%) ou pós-graduação *stricto sensu* (27,6%), oriundos de escola pública (64,7%), com estudo de língua estrangeira (88%), mas não fluentes (73,1%).

### Validação e normatização da escala

Para a análise de validação da EAPAC – expandida, aplicou-se a análise fatorial exploratória para verificar o agrupamento possível das variáveis em fatores (domínios). Foram analisados os itens Q1 até Q23; portanto, 23 itens. Estes passaram por duas rodadas de agrupamentos, seguindo o método de componentes principais e rotação *Varimax*. A partir disso, observou-se que cinco itens precisavam ser retirados, pois não tiveram correlação com os domínios da escala; ou seja, apresentaram correlação inferior a 0,5. Após a extração dos itens, o instrumento apresentou a versão final composta por 18 questões e 5 domínios (Tabela 1).

**Tabela 1:** Matriz de componente rotativa

**Questão**	**Domínio 1**	**Domínio 2**	**Domínio 3**	**Domínio 4**	**Domínio 5**
Q1	0,057	0,310	0,726	0,055	-0,022
Q2	0,104	0,095	0,615	0,056	0,236
Q3	0,055	0,115	0,783	0,158	0,033
Q4	-0,044	0,228	0,222	0,656	-0,023
Q5	0,160	0,671	0,206	0,070	0,071
Q7	0,115	0,537	0,191	0,106	0,051
Q8	0,026	0,779	0,176	-0,060	0,002
Q9	0,734	0,053	0,124	0,004	-0,008
Q10	0,727	0,150	0,023	-0,016	0,052
Q11	0,702	0,012	0,000	0,047	-0,003
Q12	0,732	0,095	0,083	0,136	0,004
Q15	0,093	-0,009	0,102	0,685	-0,069
Q16	0,133	0,046	-0,037	0,673	0,234
Q18	0,098	0,094	0,084	0,254	0,584
Q19	0,005	-0,088	0,129	-0,178	0,677
Q20	0,029	0,226	0,009	0,058	0,543
Q21	0,207	0,541	-0,072	0,195	0,218
Q22	0,568	0,186	0,027	0,087	0,169

Os cinco fatores juntos explicam 50,64% da variabilidade da amostra. Considerando que a resposta “sim” equivale a um ponto e a resposta “não” a zero ponto, o domínio 1 tem o máximo de 5 pontos, o domínio 2, o máximo de 4 pontos, os domínios 3, 4 e 5, o máximo de 3 pontos cada um. O Quadro 1 mostra a composição da EAPAC-expandida após a análise de validação.

**Quadro 1:** EAPAC – expandida após a validação

**EAPAC - ESCALA DE AUTOPERCEPÇÃO DE HABILIDADES DO PROCESSAMENTO AUDITIVO CENTRAL - EXPANDIDA**			
Data:_____/_____/_________			
Nome:____________________________________________			
Idade:____________________ Escolaridade:_____________________________________________________			
Telefone: ( ) ___________ - ____________			
Você fez o ensino médio em ( ) escola pública ( ) escola particular ( ) não se aplica			
Você é fluente em alguma língua estrangeira? ( ) sim ( ) não			
Você estuda ou estudou alguma língua estrangeira? Se sim, qual e por quanto tempo?			
**QUESTÕES**		**RESPOSTAS**	
		**Sim**	**Não**
		**PONTUAÇÃO**	
		(**1**)	(0)
Q1	Você acredita ter problemas na detecção do som (som em geral, fala ou outros sons)?		
Q2	Você acredita ter problemas na localização e lateralização da fonte sonora (saber de onde chamam quando à distância, por exemplo)?		
Q3	Você acredita ter problemas na identificação dos sons em geral?		
Q4	Você acredita ter problemas na discriminação do som (diferenciar sons da fala; por exemplo, ouvir S e Z)?		
Q5	Você acredita ter problemas na atenção seletiva e sustentada ao som (ouvir e entender a fala do professor, mesmo que haja outras conversas na sala ou ruído externo, por exemplo)?		
Q6	Você acredita ter dificuldades para perceber os sons no tempo? Por exemplo, entender alguém que fala muito rápido ou que articula as palavras sem clareza.		
Q7	Você acredita ter dificuldades para ouvir e entender a fala em situações ruidosas? Por exemplo, conversando no ponto de ônibus, em restaurantes etc.		
Q8	Você apresenta ou já apresentou dificuldades acadêmicas relacionadas à concentração em algum momento da sua vida acadêmica ou durante a atividade profissional?		
Q9	Você apresenta ou já apresentou dificuldades acadêmicas relacionadas à memória em algum momento da sua vida acadêmica ou durante a atividade profissional?		
Q10	Você apresenta ou já apresentou dificuldades acadêmicas relacionadas a planejamento em algum momento da sua vida acadêmica ou durante a atividade profissional?		
Q11	Você apresenta ou já apresentou dificuldades acadêmicas relacionadas à aprendizagem em algum momento da sua vida acadêmica ou durante a formação profissional?		
Q12	Você apresenta trocas de letras que representam sons semelhantes, na escrita ou leitura? A seguir são apresentadas algumas letras que representam sons semelhantes: B – P, D – T, G – C/Q/K, V – F, Z – S, J – X/CH. Exemplo: Cola – Gola, Já – Chá, Vaca – Faca		
Q13	Você apresenta dificuldade para fazer e perceber as pausas no texto, devido aos sinais de pontuação?		
Q14	Você apresenta dificuldade para perceber quando a pessoa quer dar um significado diferente à informação dita realizando modificação no seu tom de voz?		
Q15	Você apresenta dificuldade para entender piadas ou palavras com duplo sentido?		
Q16	Você apresenta dificuldade para perceber e reproduzir ritmos?		
Q17	Se você estiver conversando com alguém e não ouvir um trecho que a pessoa disse, tem dificuldade de compreender toda a mensagem/fala do outro?		
Q18	Você apresenta dificuldade em finalizar atividades complexas (que exigem formulação e execução de respostas) no prazo estipulado?		

Após a validação do instrumento, foram analisados os valores de sensibilidade e especificidade, e foi definido o ponto de corte considerando a presença de TPAC quando uma habilidade auditiva se mostrava alterada (TPAC 1 teste) e quando havia a alteração em duas ou mais habilidades auditivas (TPAC 2 ou mais testes). Também foram considerados os resultados da avaliação do PAC por processos auditivos. Foram consideradas as pontuações por domínio e a pontuação total obtida na escala, portanto, não foram consideradas questões isoladas. Os dados foram apresentados na Tabela 2 e as curvas ROC, na Figura 1.

**Tabela 2:** Dados normativos da EAPAC-expandida

**Variáveis padrão-ouro**	**EAPAC-expandida**		**Área**	**valor *p***	**IC 95%**		**Ponto de corte**	**Sensibilidade**	**Especificidade**
**TPAC 1 teste**	**Domínio1**		0,528	0,664	0,394	0,661	2,5	59,6%	48,1%
	**Domínio2**		0,483	0,784	0,363	0,602	2,5	39,3%	66,7%
	**Domínio3**		0,557	0,369	0,440	0,674	0,5	47,2%	59,3%
	**Domínio4**		0,523	0,717	0,398	0,648	0,5	28,1%	77,8%
	**Domínio5**		0,603	0,107	0,490	0,715	0,5	42,7%	74,1%
	**Escala total**		0,542	0,509	0,417	0,667	4,5	65,2%	40,7%
**TPAC 2 ou mais testes**	**Domínio1**		0,552	0,342	0,446	0,657	2,5	62,0%	45,5%
	**Domínio2**		0,433	0,221	0,328	0,539	2,5	38,0%	62,1%
	**Domínio3**		0,517	0,755	0,408	0,626	0,5	44,0%	53,0%
	**Domínio4**		0,523	0,666	0,417	0,630	0,5	30,0%	75,8%
	**Domínio5**		0,557	0,295	0,450	0,664	0,5	44,0%	65,2%
	**Escala total**		0,534	0,531	0,429	0,640	5,5	54,0%	48,5%
**Processamento temporal**		Domínio1	0,420	0,138	0,315	0,525	2,5	50,9%	36,5%
		Domínio2	0,560	0,265	0,455	0,665	1,5	62,3%	46,0%
		Domínio3	0,490	0,846	0,384	0,595	0,5	45,3%	54,0%
		Domínio4	0,431	0,199	0,326	0,535	0,5	18,9%	66,7%
		Domínio5	0,425	0,167	0,321	0,529	0,5	32,1%	55,6%
		Escala total	0,456	0,418	0,350	0,562	6,5	47,2%	55,6%
**Interação binaural**		Domínio1	0,494	0,926	0,363	0,624	2,5	57,0%	39,1%
		Domínio2	0,436	0,341	0,311	0,560	2,5	35,5%	52,2%
		Domínio3	0,439	0,363	0,301	0,576	0,5	44,1%	47,8%
		Domínio4	0,516	0,817	0,388	0,644	0,5	26,9%	73,9%
		Domínio5	0,432	0,314	0,303	0,561	0,5	35,5%	47,8%
		Escala total	0,450	0,463	0,319	0,582	5,5	52,7%	57,8%
**Escuta dicótica**		Domínio1	0,394	0,212	0,227	0,561	2,5	56,3%	30,8%
		Domínio2	0,468	0,707	0,271	0,665	2,5	35,9%	46,2%
		Domínio3	0,460	0,636	0,296	0,623	0,5	44,7%	46,2%
		Domínio4	0,556	0,509	0,395	0,717	0,5	28,2%	84,6%
		Domínio5	0,531	0,716	0,360	0,702	0,5	39,8%	69,2%
		Escala total	0,446	0,526	0,279	0,613	4,5	64,1%	38,5%
**Teste de fala monoaural de baixa redundância**		Domínio1	0,560	0,288	0,452	0,668	2,5	61,8%	50,0%
		Domínio2	0,595	0,094	0,484	0,705	1,5	63,2%	52,5%
		Domínio3	0,525	0,653	0,409	0,642	0,5	50,0%	62,5%
		Domínio4	0,497	0,951	0,386	0,607	0,5	26,3%	72,5%
		Domínio5	0,439	0,284	0,327	0,552	0,5	35,5%	50,0%
		Escala total	0,543	0,450	0,434	0,652	3,5	77,6%	70,0%

**Legenda:** IC = intervalo de confiança, valor *p* ≤ 0,05

**Figura 1:** Curvas ROC: diagnóstico de TPAC por quantidade de teste alterado e processos auditivos

**Legenda:** TPAC = transtorno do processamento auditivo central

A mediana da pontuação total da escala foi 6 pontos, o primeiro quartil foi igual a 3 pontos e o terceiro quartil igual a 8 pontos. A pontuação mínima encontrada foi 0, e a máxima, 15 pontos.

Foi realizada a comparação entre as pontuações (total e por domínios) de 63 indivíduos que responderam à EAPAC – expandida em dois momentos, por meio do teste de *Wilcoxon*, e não foi observada diferença significante entre as respostas do teste e reteste (Tabela 3).

**Tabela 3:** Comparação dos resultados da EAPAC – expandida em dois momentos

	**Tempo 1**	**Tempo 2**	**valor *p*** ^*^
**Domínio 1**	3 (1 – 4)	2 (1 – 4)	0,713
**Domínio 2**	2 (1 – 3)	2 (1 – 3)	0,464
**Domínio 3**	0 (0 – 1)	0 (0 – 1)	0,928
**Domínio 4**	0 (0 – 1)	0 (0 – 0)	0,207
**Domínio 5**	0 (0 – 1)	1 (0 – 1)	0,487
**Escala total**	6 (3 – 9)	6 (4 – 9)	0,984

* Teste Wilcoxon. Dados apresentados como mediana e quartis

### Queixas autorreferidas e avaliação cognitiva e audiológica

As queixas autorreferidas mais frequentes foram: dificuldade acadêmica relacionada à concentração (63,7%), planejamento (61,8%) e memória (60,3%), dificuldade para ouvir e entender a fala em ambientes ruidosos (58,6%), perceber o som ao longo do tempo (48,6%) e atenção ao som (47,1%).

Sobre os resultados da avaliação audiológica, 86,2% dos indivíduos tiveram reflexos estapedianos contralaterais presentes em três ou quatro das frequências avaliadas na orelha direita e esquerda. A mediana dos limiares audiométricos de 0,5 a 4 kHz foi igual a 10 dBNA bilateralmente e os valores mínimos e máximos foram iguais a 0 e 20 dBNA bilateralmente. Já as medianas dos limiares audiométricos de 9 a 16 kHz foram iguais a 8,3 dBNA na orelha direita e 9,1 dBNA na orelha esquerda, sendo os valores mínimos e máximos iguais a 2,5 e 20 dBNA na orelha direita e 2,5 e 21 dBNA na orelha esquerda.

Quanto à avaliação neuropsicológica, a maioria da amostra apresentou desempenho adequado nas habilidades cognitivas. Portanto, nenhum indivíduo apresentou alterações significativas em diversas habilidades, de forma que comprometesse as avaliações realizadas. Por esse motivo, nenhum participante precisou ser excluído com base nesses resultados.

Aqueles que apresentaram diagnóstico de transtorno psiquiátrico e/ou neurológico foram excluídos durante a seleção da amostra.

Quanto à frequência de diagnóstico de TPAC, 76,7% da amostra apresentou o transtorno quando pelo menos uma habilidade auditiva se apresentava alterada na avaliação. Considerando a ocorrência de alteração no PAC quando dois ou mais testes apresentaram resultados abaixo dos valores de referência, 43,1% dos indivíduos receberam o diagnóstico de TPAC.

Sobre os resultados da avaliação do PAC por processos auditivos, 52,6% obtiveram resultados normais para os testes de processamento temporal, 80,2% no teste de interação binaural, 88,8% no teste de escuta dicótica e 66,4% no teste de fala monoaural de baixa redundância. Assim, os testes do processamento temporal e de fala monoaural de baixa redundância apresentaram maior frequência de resultados alterados.

## DISCUSSÃO

O TPAC pode ocorrer em adultos com audição normal, dificultando a comunicação e o desempenho em diversas áreas da vida^(1-3,5)^. Portanto, é necessário investigar o TPAC nesse público e os questionários apontam as dificuldades diárias enfrentadas^(5,7,23)^. Devido à carência de instrumentos elaborados para adultos brasileiros^(24)^, este estudo teve o objetivo de validar e normatizar a EAPAC-expandida.

O processo de validação seguiu os critérios recomendados na literatura, como a validade de conteúdo, verificada por meio da análise de juízes, validade de critério, por meio da comparação dos dados da EAPAC-expandida com as medidas consideradas padrão-ouro, e a validade de construto, por meio da análise fatorial^(25)^.

A análise fatorial exploratória foi utilizada para verificar os padrões dos dados em conjunto, identificando, assim, correlação entre as variáveis do instrumento. A investigação levou à exclusão de cinco questões com carga fatorial baixa, portanto inferior a 0,5, o que significa que essas questões não convergiam para um ponto em comum, e dessa forma, não apresentavam correlação. As demais questões apresentaram correlações com valores próximos ou superiores aos estabelecidos na literatura (iguais ou superiores a 0,7)^(25)^.

Após a verificação da validade convergente, identificaram-se cinco domínios na escala, diferente da versão não expandida que tem apenas dois, auditivo e acadêmico^(13)^. Portanto, as questões adicionadas ao instrumento possibilitaram a identificação de diversas habilidades auditivas e cognitivas investigadas pela escala e o agrupamento das questões a partir desse dado.

Os domínios foram definidos com base em questões sobre o desempenho acadêmico (domínio 1), atenção e compreensão da fala em condições desfavoráveis (domínio 2), percepção, localização e identificação sonora (domínio 3), discriminação sonora e identificação de *gaps* (domínio 4) e entonação e ritmo (domínio 5). Juntos, esses domínios explicaram 50,64% de variabilidade da amostra.

A variabilidade, relacionada à influência da característica e do tamanho da amostra sobre os fatores analisados, apresentou resultados equivalentes aos obtidos na avaliação psicométrica do instrumento APDQ, cujos valores foram considerados satisfatórios. No estudo mencionado, o questionário demonstrou uma variância de 50,3% ao contemplar os três domínios que o integram^(11)^.

Os itens excluídos se relacionavam à memória de curta duração e habilidades linguísticas. Ao analisar os itens que se correlacionaram aos domínios, observou-se a permanência de questões sobre dificuldade de aprendizagem e com maior envolvimento de habilidades auditivas^(26)^.

Após validar o instrumento, foram criadas curvas ROC para analisar a sensibilidade, especificidade e ponto de corte do instrumento. A análise estatística para a normatização da escala foi conduzida levando em conta múltiplas variáveis. Assim, para a construção da curva ROC, utilizaram-se como padrão-ouro: o diagnóstico de TPAC com alteração em um teste comportamental (ou seja, presença de uma habilidade alterada), ocorrência de dois ou mais testes alterados e o resultado da avaliação de cada processo auditivo individualmente. No total, foram consideradas seis variáveis, cujos dados foram apresentados separadamente.

Espera-se que a área sob a curva tenha valor mínimo de 70% e valor *p* significativo, contudo, as áreas sob as curvas foram abaixo da referência e sem significância estatística, indicando baixa sensibilidade e especificidade e pontos de corte frágeis.

Considerando a presença do TPAC devido à alteração em um teste comportamental do PAC, a escala apresentou sensibilidade de 65,2% e especificidade igual a 40,7%, com pontuação de corte igual a 4,5. Com dois ou mais testes de PAC alterados, o instrumento identificou 54% dos indivíduos com TPAC e 48,5% de quem não tinha transtorno, com pontuação mínima igual a 5,5.

A análise das propriedades da escala por processos auditivos, verificou sensibilidade e especificidade iguais a 47,2% e 55,6%, respectivamente, para o processamento temporal, 52,7% e 57,8% para o teste de interação binaural, 64,1% e 38,5% para a avaliação da escuta dicótica e 77,6% e 70% para o teste de fala monoaural de baixa redundância. As pontuações de corte foram 6,5 pontos para alteração no processamento temporal, 5,5 pontos para alteração no teste de interação binaural, 4,5 pontos para alteração no teste de escuta dicótica e 3,5 pontos para alteração no teste de fala monoaural de baixa redundância.

O estudo inicial da EAPAC mostrou pontuações de 5 e 6 para indicar alteração nos testes FR e GIN, respectivamente. A sensibilidade e especificidade foram 74% e 56% para o teste FR, e 62% e 51% para o teste GIN^(13)^. Comparando com a EAPAC-expandida, verificou-se que os valores se diferenciaram pouco em relação às medidas psicométricas da EAPAC.

As pontuações dos domínios do APDQ – Processamento Auditivo, Déficit de Atenção e Desordem de Linguagem – possibilitaram a correta classificação de mais de 80% dos grupos participantes do estudo (sem alteração clínica, alteração no PAC, TDAH e distúrbio de linguagem), conforme as recomendações da literatura especializada. A maioria dos valores de corte identificados apresentou sensibilidade e especificidade superiores a 80%^(11)^. Além disso, avaliação prévia desse instrumento em adultos indicou alta sensibilidade, atingindo 85,7%^(12)^. Ao comparar os resultados obtidos por meio do instrumento com aqueles provenientes da análise EAPAC-expandida, observa-se uma diferença entre ambas, sendo que a EAPAC-expandida apresentou valores abaixo do que é recomendado pela literatura.

Apesar das medidas psicométricas da versão expandida da EAPAC serem pouco robustas para identificar o risco para o TPAC, a escala mostrou-se útil para analisar as dificuldades auditivas apresentadas no ambiente naturalístico, portanto o impacto na vida do sujeito, auxiliando o avaliador na compreensão do caso, na elaboração de estratégias para minimizar as dificuldades apresentadas e no monitoramento da evolução clínica.

Em relação à pontuação da escala, as dificuldades autorreferidas receberam um ponto; assim, quanto maior for a pontuação, mais queixas autorrelatadas. A amostra apresentou pontuação mediana igual a 6 pontos, corroborando o estudo de Abreu e colaboradores^(13)^, no qual também se observou pontuação média igual a 6,1.

Ao comparar o comportamento auditivo ao longo do tempo, não foram verificadas modificações significativas. Mudanças na via nervosa auditiva ocorrem em função do avanço da idade, devido às perdas neuronais que se iniciam entre 35 e 40 anos, causando queixas auditivas, como alteração na percepção da fala^(3)^, o que não foi identificado na amostra, uma vez que não houve alteração na frequência de queixas autorreferidas, pois a idade mediana da amostra é inferior à idade propensa a essas perdas.

Por meio da EAPAC-expandida, foi possível identificar que os itens mais frequentemente assinalados, portanto, as queixas mais prevalentes, foram: a dificuldade de aprendizado devido à concentração, planejamento e memória, além de dificuldade para compreender a fala na presença de ruído, perceber ao som no tempo e manter a atenção ao som.

O processo de aprendizagem é influenciado por habilidades cognitivas, como a atenção, a memória, as funções executivas, dentre outras, que ajudam a organizar e a integrar informações, levando à mudança de comportamento e à compreensão de novos conceitos. Com o tempo escolar, as tarefas se tornam mais complexas, requerendo mais aporte cognitivo. Déficits nessas habilidades, especialmente a atenção, memória e planejamento, essenciais também para o PAC, podem causar problemas de aprendizagem^(27)^, como evidenciado na pesquisa.

Essas habilidades cognitivas são importantes para o PAC, pois direcionam o foco para o estímulo auditivo, inibem os distratores e promovem o armazenamento e interpretação da informação para gerar respostas corretas ao estímulo auditivo^(2,4)^.

Conforme a literatura, indivíduos com prejuízos na memória, função executiva e atenção apresentam dificuldade para identificar a fala em ambientes ruidosos^(2)^, que, somada às alterações nas habilidades auditivas, causam queixas relacionadas à atenção sonora e identificação do som^(7,28,29)^.

Estudos frequentemente reportam queixas relacionadas à dificuldade para entender o som na presença de ruídos. Na pesquisa de Cancel e colaboradores^(6)^, 44% dos adultos com idade mediana de 35 anos e audição normal relataram a sensação de perda auditiva ou dificuldade auditiva na presença de ruído. O estudo de Pereira e colaboradores^(14)^ também identificou alto percentual de queixa auditiva relacionada à compreensão da fala no ruído (56,8%).

Pesquisas realizadas com crianças e adolescentes também identificam tais sintomas. No estudo conduzido por O’Hara e Mealings^(11)^, o instrumento APDQ evidenciou que indivíduos com alterações no PAC apresentam dificuldades acentuadas para compreender a fala em ambientes ruidosos, escuta durante a realização de multitarefas e ao escutar interlocutores com articulação pouco clara. Participantes diagnosticados com TDAH demonstraram desafios principalmente relacionados à atenção, memória auditiva e maior suscetibilidade a distrações causadas pelo ruído ambiente. Os sintomas mapeados pelo APDQ revelaram similaridade com as dificuldades apontadas pela EAPAC-expandida.

Perceber, identificar e reconhecer o som demanda habilidades auditivas e cognitivas em comum, o que justifica as dificuldades auditivas autorrelatadas frequentes na presente pesquisa. Ademais, os resultados obtidos mostraram consonância com os dados observados na literatura.

A frequência de TPAC na amostra foi alta, tanto quando um único teste comportamental estava alterado quanto quando dois ou mais mostravam alteração. A prevalência do TPAC não está bem definida, mas a literatura sugere a ocorrência entre 0,5% e 1%, aumentando quando ocorre com outros fatores^(30)^.

Os resultados do presente estudo corroboraram a literatura^(6)^, que também mostrou o percentual elevado do TPAC. Segundo os autores, 51% da amostra apresentou o TPAC. A alta prevalência no estudo sobre a EAPAC-expandida, pode estar ligada ao fato que a maioria dos sujeitos que manifestaram o desejo de participar da pesquisa apresentavam queixas auditivas e, portanto, sugestivo de TPAC, o que pode ter influenciado os resultados desta pesquisa, sendo necessários novos estudos com sujeitos sem TPAC.

Segundo a Academia Brasileira de Audiologia^(21)^, um único teste comportamental alterado é suficiente para o diagnóstico de TPAC, o que pode elevar a prevalência do transtorno. Tendo em vista a ocorrência do TPAC na população geral e a interferência de fatores cognitivos e linguísticos no desempenho auditivo e o diagnóstico inadequado do transtorno^(30)^, é importante discutir e definir os critérios para o diagnóstico do TPAC.

Quanto à avaliação do PAC, a maioria dos testes apresentou resultados normais, mas o processamento auditivo temporal apresentou mais alterações, seguido do teste de fala monoaural de baixa redundância. Os dados concordaram parcialmente com a literatura, que apontou déficits na escuta dicótica (integração binaural) e no teste de fala monoaural de baixa redundância (fala no ruído) dentre os indivíduos adultos encaminhados para a avaliação do PAC devido à queixa auditiva^(6)^.

A percepção de variações no som em função do tempo envolve as habilidades auditivas de ordenação e resolução temporal, que compõem o processamento auditivo temporal. Essas habilidades auxiliam na compreensão da fala e no reconhecimento dos fonemas e variações dos aspectos suprassegmentais da fala; ou seja, perceber e identificar as características do som ao longo do tempo^(31,32)^.

A literatura mostrou que homens jovens detectam melhor os *gaps* do que as mulheres^(31)^, sendo que aqueles com o TPAC apresentaram piores resultados nos testes de ordenação e de resolução temporal auditiva^(31)^. Portanto, o percentual elevado de alteração no processamento auditivo temporal na presente pesquisa pode ser justificado pela maioria de mulheres e percentual elevado de TPAC na amostra.

Quando a fala é apresentada em boas condições sonoras e somada aos fatores extrínsecos, como o contexto, familiaridade com as palavras e o tema do discurso, as regras da língua, dentre outras, é mais fácil compreendê-la. No entanto, em condições ruins de escuta, a compreensão da fala torna-se uma situação desafiadora, que demanda o maior uso de recursos cognitivos e auditivos (recursos intrínsecos) para que o reconhecimento da fala seja realizado^(31)^.

A escuta de palavras monossilábicas na presença de ruído foi difícil para a amostra estudada. A dificuldade para compreender a fala na presença de ruído foi uma das queixas comuns autorrelatadas na escala expandida e identificada no teste comportamental. Assim, as palavras curtas e, portanto, com poucas pistas auditivas e baixa redundância, foram difíceis de serem reconhecidas, mesmo sendo palavras familiares^(31,33)^. O estudo de Turcatto et al.^(7)^ também mostrou que adultos com e sem TPAC tiveram dificuldade na tarefa de fala monoaural de baixa redundância com palavras monossilábicas.

O presente estudo contribuiu para as investigações sobre a EAPAC-expandida, destacando sua utilidade para compreender o impacto das dificuldades para analisar o estímulo sonoro em tarefas do cotidiano em adultos, dada a falta de ferramentas adequadas. Contudo, a pesquisa teve limitações, como a ausência de investigação da influência de tarefas que ampliam o desempenho auditivo, como o treinamento musical, nas respostas dos sujeitos e uma amostra menor do que a desejada. Outro aspecto relevante para análise refere-se à apuração da influência das questões associadas à aprendizagem, que podem estar vinculadas a componentes não auditivos ou cognitivos, nos resultados obtidos na validação do instrumento.

Sugere-se que futuros estudos considerem a inclusão de grupo controle constituído por indivíduos com desempenho auditivo e cognitivo dentro dos parâmetros de normalidade. Além disso, destaca-se a importância de que a aplicação da EAPAC-expandida seja conduzida diretamente pelo fonoaudiólogo, ao invés de ser autoadministrada pelo participante sem supervisão profissional, visando à adequada comparação dos resultados obtidos nas duas modalidades de aplicação.

## CONCLUSÃO

Embora a EAPAC-expandida não tenha apresentado, nos processos para validação e normatização, parâmetros psicométricos adequados para a identificação de indivíduos de risco para o TPAC, ela mostrou ser um instrumento útil para investigar as dificuldades enfrentadas pelo indivíduo no ambiente naturalístico. O instrumento se baseia no autorrelato de queixas, portanto apresenta a relevância de apontar o impacto do problema na vida do sujeito. A escala é capaz, assim, de nortear o avaliador na definição de estratégias que podem minimizar as queixas autorreferidas e na compreensão do impacto das dificuldades relatadas. Dessa forma, a EAPAC- expandida pode complementar a bateria de avaliação do PAC como um instrumento que permite identificar a autopercepção auditiva do indivíduo.
